# Numerical Approach for the Assessment of Micro-Textured Walls Effects on Rubber Injection Moulding

**DOI:** 10.3390/polym13111739

**Published:** 2021-05-26

**Authors:** María García-Camprubí, Carmen Alfaro-Isac, Belén Hernández-Gascón, José Ramón Valdés, Salvador Izquierdo

**Affiliations:** Instituto Tecnológico de Aragón (ITAINNOVA), C/María de Luna, n°7-8, 50018 Zaragoza, Spain; calfaro@itainnova.es (C.A.-I.); bhernandez@itainnova.es (B.H.-G.); jrvaldes@itainnova.es (J.R.V.); sizquierdo@itainnova.es (S.I.)

**Keywords:** rubber, seals, injection moulding, surface texture, modelling, computational fluid dynamics, reduced order model

## Abstract

Micro-surface texturing of elastomeric seals is a validated method to improve the friction and wear characteristics of the seals. In this study, the injection process of high-viscosity elastomeric materials in moulds with wall microprotusions is evaluated. To this end, a novel CFD methodology is developed and implemented in OpenFOAM to address rubber flow behaviour at both microscale and macroscale. The first approach allows analyzing the flow perturbation induced by a particular surface texture and generate results to calculate an equivalent wall shear stress that is introduced into the macroscale case through reduced order modelling. The methodology is applied to simulate rubber injection in textured moulds in an academic case (straight pipe) and a real case (D-ring seal mould). In both cases, it is shown that textured walls do not increase the injection pressure and therefore the manufacturing process is not adversely affected.

## 1. Introduction

In industry, environmentally friendly processes are presently a must for a greener future. Within the broad industry concept, elastomers are one of the most versatile engineering materials and one of their main engineering applications is in the production of dynamic seals. Although they are generally regarded as high volume, low cost components, elastomeric dynamic sealing elements are a critical part of rotating and sliding devices in diverse industries such as manufacturing, automotive, aerospace, energy, power generation and construction since they must prevent any kind of leakage between the domains they separate, enduring millions of cycles under often harsh operational conditions. While they have a very important function, the problem with seals is that friction generates heat, accelerates wear and causes premature damage and leakage, shortening the seals lifetime, as well as increasing the energy consumption of the systems [1,2]. According to the European Network for Industrial Wear Prevention, estimates show that between 2 and 4 percent of an industrialised country’s gross domestic product (GDP) is lost through friction and mechanical parts wearing out. Within this background, there is a strong requirement to increase the efficiency of elastomeric dynamic seals by reducing their friction.

Recent innovative approaches aim at producing high performance seals with no addition of new raw material and with a cost-effective and easy-to-integrate mass production approach of textured elastomeric seals [3]. Surface texturing of the seals will improve the friction and wear characteristics of the seals without compromising the inherent mechanical properties of the seals or altering the bulk material chemistry. The innovative production process consists of applying the texture patterns to the mould surface by laser. The texture is then transferred to the rubber component surface via the mould tool during the injection process, the most used technology of polymer processing at present. However, there is an initial uncertainty about how the presence of micro-protrusions affects the injection process with elastomeric materials that have very high viscosity. To that end, using numerical simulation of the injection moulding process, it is possible to predict the quality of moulded part and the injection time and to detect the potential problems at the early design stage. Moreover, by means of moulding process simulation at micro scale, the behaviour of the injected material over the mould wall protrusions can be assessed, analyzing how the material flows around the mould texture.

Computer-aided engineering (CAE) simulation technologies based on computational fluid dynamics (CFD) are extensively used by injection moulders to predict and optimise the mould design and operational conditions for the injection process, because trial and error analysis based on industrial pilot lines would be unaffordable in terms of both time and costs. Likewise, numerical tools can be used to virtually evaluate the material response without the physical use of the real material and without compromising the mould manufacturing and the available time for production. Additionally, CFD studies provide an efficient tool to predict, and hence avoid re-designing the mould before it is actually made, distinct problems that may appear when the mould is under operation such weld lines, sink marks or air trapped inside the mould, among others. Therefore, it is possible to minimize mistakes and costs before the mould production. In this regard, numerous computational studies of injection processes can be found in the literature [4,5,6]. Nevertheless, despite the inherent advantages, the use of computational simulation is not widespread throughout all companies but its use is inhibited by the cost of the software licence. As reported in the literature, some of the most commonly used commercial software are Moldex 3D [7], Ansys Fluent [8] and Moldflow [9], among others. In contrast, the OpenFoam software is an attractive open source CFD tool where the user can implement any physical phenomena and material behaviour law [10]. Despite the fact that some fluid dynamic phenomena are described and documented publicly [11], to the authors knowledge, there is no available code for simulating advanced rheological behaviour of rubber materials.

The increasing demand of micro-components has motivated many CFD studies in the literature that are focused on micro injection moulding [12,13,14,15]. This numerical approach allows the manufacturing of high precision components, where micro-scale phenomena related to the operational conditions occur. Specifically, interactions between the surface roughness and the polymer interfacial tension provoke critical interactions that could lower the effectiveness of the process. In this case, the simulation of micro-injection processes allows analyzing them at the micro-scale. Additionally, the manufacturing of high-performance micro-texture seals couples phenomena at both the micro-scale, where the micro-protrusions are relevant for the study of specific wall effects, and the macro-scale, where the mould filling is strongly affected by the operational conditions from the injection process. Commercial softwares specifically developed for mould filling of thermoplastic materials and elastomers, such as the above-mentioned Moldex3D or Moldflow, are widely used. Their main advantage is the short computing times, which comes at the cost of simplificating the governing equations of the flow [16,17]. Therefore the accuracy of the multiphysic model is not assured and its applicability to study small perturbations of the flow, such those produced by the mould wall texture, can be highly questionable. Another limitation of commercial software is their constrained access to source code variables. Thus, the implementation of the wall law to include the microscale effects on the macroscale flow as described in this work is not possible. Difficulties to numerically simulate injection moulding of rubber components with micro-features using commercial packages are found in the literature [18,19,20]. Therefore, there is a need to tackle a numerical scenario for the specific problem of micro-textured elastomeric seals that can reveal whether undesirable effects come from the micro-protrusions on the mould surface.

Within this background, the promising technology of micro-surface texturing of elastomeric seals for friction reduction motivates the current paper whose goal is to assess the behaviour of the injected material over the mould wall protrusions, in order to see how the moulded material will conform to the mould texture. In this research, a novel numerical methodology implemented on open source software is developed to analyze the injection process of elastomers in micro-textured moulds considering effects at both the micro and the macro scales. The microscale approach pursues the knowledge generation regarding the flow perturbations induced by the presence of mould wall protrusions in the range of tens of microns. In contrast, the macro-scale approach is aimed at the CFD simulation of the injection in rubber moulds. To link the results from both approaches, the microscale results are used to generate a reduced order model (ROM), which is introduced in the macroscale simulation as a boundary law in the textured walls. The outcome from this investigation will definitely contribute to increasing the knowledge of the industrial sector, which is strongly interested in building a greener future by improving the efficiency of the manufacturing processes of high-performance components.

## 2. Materials and Methods

This section consists of three main subsections. First, Section 2.1 presents the governing equations of the phenomena involved in the rubber flow during the injection processs. Next, Section 2.2 introduces the fundamentals of the wall law approach developed to include the micro-texture wall effects on the dynamics of the microscale flow. Finally, in Section 2.3, it is described the numerical method to solve the mathematical model described in former Section 2.1 and Section 2.2.

### 2.1. Rubber Injection Moulding: Mathematical Model

The governing equations of the rubber flow, during rubber injection moulding, are presented in this section. The physics involved in this process are, namely: (i) multiphase flow with interface tracking; (ii) mass conservation; (iii) momentum conservation; (iv) curing; and (v) heat transfer.

#### 2.1.1. Alpha Equation

Mass conservation equation of the primary phase:

(1) $$\frac{\partial \alpha_{1}\rho_{1}}{\partial t}+\nabla \cdot \left(\alpha_{1}\rho_{1}\vec{v}\right)=0$$

where $\alpha_{1}$, $\rho_{1}$ and $\vec{v}$ are the volume fraction, the density and the velocity of the primary phase, respectively. The velocity subscript is omitted since two-phase flows such as the one under study (immiscible no slip phases) allow us to use a single momentum equation and velocity field to model both phases [21].

To ensure continuity, the secondary phase volume fraction must satisfy:

(2) $$\alpha_{2}=1-\alpha_{1}$$

both volume fractions taking values between 0 and 1, according to:

(3) $$\alpha_{1}=\left\{\begin{array}{cc} 1, & \;\mathrm{for}\;a\;\mathrm{point}\;\mathrm{inside}\;\mathrm{the}\;\mathrm{primary}\;\mathrm{phase}.\\ 0<\alpha_{1}<1, & \;\mathrm{for}\;a\;\mathrm{point}\;\mathrm{at}\;\mathrm{the}\;\mathrm{interface}.\\ 0, & \;\mathrm{for}\;a\;\mathrm{point}\;\mathrm{inside}\;\mathrm{the}\;\mathrm{secondary}\;\mathrm{phase}. \end{array}\right.$$

Expanding Equation (1), dividing it by $\rho_{1}$, applying the chain rule to the derivatives of density and rearranging:

(4) $$\frac{\partial \alpha_{1}}{\partial t}+\nabla \cdot \left(\alpha_{1}\vec{v}\right)+\frac{\alpha_{1}}{\rho_{1}}\frac{\partial \rho_{1}}{\partial p}\frac{Dp}{Dt}=0$$

Assuming linear equations of state:

(5) $$\left(\frac{\partial \rho_{1}}{\partial p}\right){|}_{s}=\psi_{1}=constant$$

where $\psi_{1}$ is the isentropic (adiabatic and reversible) compressibility of the primary phase, it is fulfilled that:

(6) $$\frac{D\rho_{1}}{Dt}=\psi_{1}\frac{Dp}{Dt}$$

Replacing (5) and (6) into (4), it results in:

(7) $$\frac{\partial \alpha_{1}}{\partial t}+\nabla \cdot \left(\alpha_{1}\vec{v}\right)+\frac{\alpha_{1}}{\rho_{1}}\frac{D\rho_{1}}{Dt}=0$$

For convenience, the term $-\alpha_{1}\nabla \cdot \vec{v}$ is added to both sides of Equation (7):

(8) $$\frac{\partial \alpha_{1}}{\partial t}+\nabla \cdot \left(\alpha_{1}\vec{v}\right)-\alpha_{1}\nabla \cdot \vec{v}+\frac{\alpha_{1}}{\rho_{1}}\frac{D\rho_{1}}{Dt}=-\alpha_{1}\nabla \cdot \vec{v}$$

replacing Equation (12) into the right-hand side of Equation (8) and rearranging:

(9) $$\frac{\partial \alpha_{1}}{\partial t}+\nabla \cdot \left(\alpha_{1}\vec{v}\right)=\alpha_{1}\alpha_{2}\left(\frac{1}{\rho_{2}}\frac{D\rho_{2}}{Dt}-\frac{1}{\rho_{1}}\frac{D\rho_{1}}{Dt}\right)+\alpha_{1}\nabla \cdot \vec{v}$$

For numerical reasons, an artificial term is added to the left-hand side of Equation (9) to counteract the numerical diffusion of the interface:

(10) $$\frac{\partial \alpha_{1}}{\partial t}+\nabla \cdot \left(\alpha_{1}\vec{v}\right)+\nabla \cdot [\alpha_{1}\alpha_{2}\vec{v_{r}}]=\alpha_{1}\alpha_{2}\left(\frac{1}{\rho_{2}}\frac{D\rho_{2}}{Dt}-\frac{1}{\rho_{1}}\frac{D\rho_{1}}{Dt}\right)+\alpha_{1}\nabla \cdot \vec{v}$$

$\vec{v_{r}}$ being a compression velocity, denoting the relative velocity of the two-fluids. This artificial-compression term is only active in the interface region, where $\alpha_{1}\left(1-\alpha_{1}\right)>0$. As such, it does not affect the solution significantly outside this region [22,23].

#### 2.1.2. Mass-Conservation Equation

The differential form of the mass conservation equation may be written as the sum of the mass conservation equations of the two phases (from Equation (4)) as:

(11) $$\nabla \cdot \vec{v}+\left(\frac{\alpha_{1}}{\rho_{1}}\frac{\partial \rho_{1}}{\partial p}+\frac{\alpha_{2}}{\rho_{2}}\frac{\partial \rho_{2}}{\partial p}\right)\left(\frac{\partial p}{\partial t}+\vec{v}\cdot \nabla p\right)=0$$

replacing Equation (5) into Equation(11), considering the definition of the substantial derivative and $\alpha_{1}+\alpha_{2}=1$, and rearranging:

(12) $$\nabla \cdot \vec{v}=-\left(\frac{\alpha_{1}}{\rho_{1}}\frac{D\rho_{1}}{Dt}+\frac{\alpha_{2}}{\rho_{2}}\frac{D\rho_{2}}{Dt}\right)$$

#### 2.1.3. Momentum-Conservation Equation

The momentum-conservation equation is, in differential and vector form:

(13) $$\frac{\partial (\rho \vec{v})}{\partial t}+\nabla \cdot (\rho \vec{v}\vec{v})=\nabla \cdot \vec{\vec{\tau }}+\vec{f_{v}}+\vec{f_{s}}$$

where $\vec{\vec{\tau }}$ is the stress tensor, and $\vec{f_{v}}$ and $\vec{f_{s}}$ stand for the body and surface forces. In this particular case, there are two external forces acting on the fluid due to gravity (body force) and surface tension (surface force) given by [24]:

(14) $$\vec{f_{v}}=\rho \vec{g}$$

(15) $$\vec{f_{s}}=\sigma \kappa \nabla \alpha_{1}$$

where $\vec{g}$ is the gravity acceleration, σ is the surface tension, and κ stands for the interface curvature:

(16) $$\kappa =-\nabla \cdot \left(\frac{\nabla \alpha_{1}}{|\nabla \alpha_{1}|}\right)$$

where $\frac{\nabla \alpha_{1}}{|\nabla \alpha_{1}|}$ is the unit normal vector at the interface, calculated using the phase fraction field. According to the constitutive relation for a Newtonian fluid, the stress tensor may then be written as:

(17) $$\vec{\vec{\tau }}=\mu [\nabla \vec{v}+{\left(\nabla \vec{v}\right)}^{T}]-\left[p+\left(\frac{2}{3}\mu -\mu_{V}\right)\nabla \cdot \vec{v}\right]\vec{\vec{I}}$$

where $\mu_{V}$ is the coefficient of bulk viscosity, μ is the dynamic viscosity of the fluid and $\vec{\vec{I}}$ the identity matrix. Considering $\nabla \cdot \vec{v}=\mathrm{tr}\left[{\left(\nabla \vec{v}\right)}^{T}\right]$ and the fact that the bulk viscosity is negligible under the operating conditions, $\mu_{V}\approx 0$ (laminar flow), the momentum conservation equation may be expressed, from Equations (13)–(15) and (17), as follows:

(18) $$\begin{array}{c} \frac{\partial (\rho \vec{v})}{\partial t}+\nabla \cdot (\rho \vec{v}\vec{v})-\nabla \cdot (\mu \nabla \vec{v})-\nabla \cdot \left\{\mu \left[{\left(\nabla \vec{v}\right)}^{T}-\frac{2}{3}\mathrm{tr}\left[{\left(\nabla \vec{v}\right)}^{T}\right]\vec{\vec{I}}\right]\right\}\\ =-\nabla p+\rho \vec{g}+\sigma \kappa \nabla \alpha_{1} \end{array}$$

For convenience [25], pressure (*p*) is replaced by the modified pressure ($p^{*}$), defined as: $p^{*}=p-\rho \vec{g}\cdot \vec{x}$. The momentum equation (Equation (18)) thus results in:

(19) $$\begin{array}{c} \frac{\partial (\rho \vec{v})}{\partial t}+\nabla \cdot (\rho \vec{v}\vec{v})-\nabla \cdot (\mu \nabla \vec{v})-\nabla \cdot \left\{\mu \left[{\left(\nabla \vec{v}\right)}^{T}-\frac{2}{3}\mathrm{tr}\left[{\left(\nabla \vec{v}\right)}^{T}\right]\vec{\vec{I}}\right]\right\}\\ =-\nabla p^{*}-\vec{g}\cdot \vec{x}\nabla \rho +\sigma \kappa \nabla \alpha_{1} \end{array}$$

This variable change is advantageous for the specification of the pressure at the boundaries of the space domain. Additionally, this treatment enables efficient numerical treatment of the steep density jump at the interface by including the term $\vec{g}\cdot \vec{x}\nabla \rho$.

In Equation (19), the rubber viscosity is described according to the “Reactive Viscosity Model”, that defines the temperature, shear rate, and cure dependence of thermoset materials ($\mu =f(\omega ,T,\dot{\gamma })$) as follows:

(20) $$\mu_{\left(\omega ,T,\dot{\gamma }\right)}=\frac{\mu_{0_{\left(T\right)}}}{1+{\left(\frac{\mu_{0_{\left(T\right)}}\dot{\gamma }}{\tau^{*}}\right)}^{1-n}}{\left(\frac{\omega_{g}}{\omega_{g}-\omega }\right)}^{\left(C_{1}+C_{2}\omega \right)}$$

where $\mu_{0_{\left(T\right)}}=Be^{\left(\frac{T_{b}}{T}\right)}$ and ω is the degree of cure (see Section 2.1.4); *n*, $\tau^{*}$, *B*, $T_{b}$, $C_{1}$, $C_{2}$, $\omega_{g}$ are data-fitted coefficients obtained from experimental characterization.

#### 2.1.4. Rubber Cure Kinetics

Rubber cure kinetics is here described according to the n-th order reaction kinetics (Kamal model), used to calculate the curing behavior of a thermoset material, given by:

(21) $$\frac{D\omega }{dt}=\left(K_{1}+K_{2}\omega^{m_{c}}\right){\left(1-\omega \right)}^{n_{c}}$$

where ω stands for the degree of cure and ranges between 0 and 1; *T* is the temperature; *t* represents the time; and the parameters $K_{1}$ and $K_{2}$ are given by:

(22) $$\begin{array}{c} K_{1}=A_{1}exp\left(-\frac{E_{1}}{T}\right)\\ K_{2}=A_{2}exp\left(-\frac{E_{2}}{T}\right) \end{array}$$

$m_{c}$, $n_{c}$, $A_{1}$, $A_{2}$, $E_{1}$, $E_{2}$ being the model parameters to be experimentally characterized.

Some materials, such is the case of rubber, undergo an induction period befores curing starts to take place. The length of this induction period is given by:

(23) $$t_{z}=B_{1}exp\left(\frac{B_{2}}{T}\right)$$

where $t_{z}$ represents the induction time; *T* is the temperature and $B_{1}$ and $B_{2}$ are the parameters to be experimentally estimated.

To take into account the induction period, Equation (21) is modified as follows:

(24) $$\frac{D\omega }{dt}=Y*\left(K_{1}+K_{2}\omega^{m_{c}}\right){\left(1-\omega \right)}^{n_{c}}$$

where Y stands for a boolean variable:

(25) $$Y=\frac{1}{2}\left(\frac{\left(t_{r}-t_{z}\right)}{|(t_{r}-t_{z})|}+1\right)=\left\{\begin{array}{c} 1\Rightarrow t_{r}>t_{z}\;\mathrm{Cure}\;\mathrm{can}\;\mathrm{start}\\ 0\Rightarrow t_{r}<t_{z}\;\mathrm{Cure}\;\mathrm{do}\;\mathrm{not}\;\mathrm{start} \end{array}\right.$$

#### 2.1.5. Energy-Conservation Equation

The equation of the total energy (e+K) conservation is given by:

(26) $$\begin{array}{cc} \frac{\partial \left[\rho \left(e+K\right)\right]}{\partial t}+ & \nabla \cdot [\rho \vec{v}\left(e+K\right)]\\ = & -\nabla \cdot (p\vec{v})+\Phi_{v}+\vec{f_{v}}\cdot \vec{v}+\vec{f_{s}}\cdot \vec{v}-\nabla \cdot \left(\lambda \nabla T\right)+Q_{rad}+Q_{reac} \end{array}$$

where *e* is the specific internal energy, $K=v^{2}/2$ is the specific kinetic energy, $\vec{q}$ is the conduction heat flux, $Q_{rad}$ and $Q_{reac}$ stand for the radiation and chemical reaction volumetric heat sources, respectively, and $\Phi_{v}$ is the viscous heat source.

Rubber is a highly viscous non-compressible fluid, where viscous dissipation effects might be of significance: $\Phi_{v}=\nabla \cdot (\vec{\vec{\tau \overset{´}{}}}\cdot \vec{v})\neq 0$. Compressibility effects on rubber are negligible but might be of relevance on the air side (secondary phase), thermal accuracy is though not of importance in the secondary phase and thus it is assumed that $\nabla \cdot (p\vec{v})\approx 0$. The curing reaction is non-isothermal; however the chemical-reaction heat-source is at the moment neglected ($Q_{reac}\approx 0$). Injection moulding temperatures are not high enough for radiation heat transfer to be relevant ( $Q_{rad}\approx 0$). Heat sources due to body and surface forces can be neglected ($\vec{f_{v}}\cdot \vec{v}\approx 0$, $\vec{f_{s}}\cdot \vec{v}\approx 0$). Equation (26) can thus be simplified as follows:

(27) $$\frac{\partial \left[\rho \left(e+K\right)\right]}{\partial t}+\nabla \cdot [\rho \vec{v}\left(e+K\right)]=\Phi_{v}-\nabla \cdot \left(\lambda \nabla T\right)$$

By definition: $de=C_{v}dT$, $C_{v}$ being the heat capacity at constant volume. Assuming $C_{v}$ is constant, then $e=C_{v}T$ and Equation (27) leads to:

(28) $$\frac{\partial \left(\rho T\right)}{\partial t}+\nabla \cdot (\rho \vec{v}T)+\frac{1}{C_{v}}\left[\frac{\partial \left(\rho K\right)}{\partial t}+\nabla \cdot (\rho \vec{v}K)\right]+\nabla \cdot \left(\frac{\lambda }{C_{v}}\nabla T\right)=\frac{\Phi_{v}}{C_{v}}$$

In Equation (28) the specific heat at constant volume $C_{v}$ of the mixture is calculated as a function of the mass fractions: $C_{v}=\left(y_{1}C_{v1}+y_{2}C_{v2}\right)$, where $y_{1}=\left(\alpha_{1}\frac{\rho_{1}}{\rho }\right)$ and $y_{2}=\left(1-y_{1}\right)$ are the mass fractions of primary and secondary phases, respectively.

### 2.2. Wall Texture: An Effective Viscosity Modelling Approach

A novel approach is here presented to include the microscale wall texture effects on the macroscopic filling model. It consists of developing a new wall law that accounts for the wall texture effects and enables the use of smooth walls geometries for the macroscale filling simulation of textured moulds.

**Figure 1:**
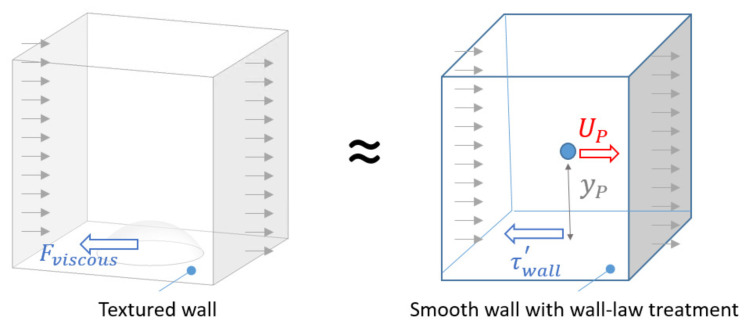
Sketch representing the concept of the effective viscosity wall law for textured walls.

Figure 1 illustrates a representative volume of fluid near the textured wall and the equivalent volume of fluid near a smooth wall. The texture effects on the rubber flow are considered in the smooth wall using an ad hoc developed wall-law, that modifies the viscosity at the wall:

(29) $$\mu_{wall_{{}_{\left(T\right)}}}^{eff}=\frac{y_{P}}{U_{P}}\tau_{wall_{\left(T\right)}}^{{}^{\prime }}$$

so that the resulting fluid-wall friction force ($F_{viscous}$) is the same than in the case of the textured wall:

(30) $$F_{viscous}=\tau_{wall_{\left(T\right)}}^{{}^{\prime }}A_{wall}$$

where $\tau_{wall_{\left(T\right)}}^{{}^{\prime }}$ is the effective viscous stress at the smooth wall for a given temperature (*T*), $U_{P}$ is the mean velocity of the flow at the center of the cell near the wall boundary, and $y_{P}$ is the distance between the wall and the center of the cell near the wall (see Figure 1). Please note that for the sake of simplicity, the effective viscosity at the wall does not depend on the cure degree, as during the filling process it is expected to be negligible due to the induction time. The overall concept of defining an effective viscosity to model particular fluid-wall interactions has been previously used in the literature for other applications [26,27].

In order to be able to model the effective viscosity at the wall, according to Equation (29), it is important to characterize the viscous stress ($\tau_{wall_{\left(T\right)}}^{{}^{\prime }}$) under different operating conditions ($U_{p},y_{P},T$). This is addressed by performing numerical simulations of the rubber flow on the textured wall and postprocessing the results using a model order reduction technique (see Section 2.3.1).

### 2.3. Numerical Approach

A new OpenFOAM solver, called rubberFoam, has been developed to solve the comprehensive mathematical model described in Section 2.1 and Section 2.2. It is based on the standard solver compressibleInterFoam for two compressible, non-isothermal immiscible fluids using a VOF (volume of fluid) phase-fraction-based interface capturing approach [10].

The rubberFoam stands thus for an evolved version of the compressibleInterFoam, being the major modifications aimed at accounting for the particular non-Newtonian behaviour of rubbers (Equation (20)) and the curing chemical reaction (Equation (24)). A new structure of classes has been developed to this end, being the most relevant ones indicated below.

A new mixture class is created (mixture(U, phi, omega)), where the thermo-physical properties of the main-phase (of a new type rhoThermoNonNewtonian) are not only dependent on the operating pressure and temperature, but also on the velocity field (U) and an auxiliary scalar field (omega); whereas the properties of the secondary-phase (of standard type rhoThermo) remain only dependent on the operating pressure and temperature.

A new thermo-package has been defined (rhoThermoNonNewtonian) to describe the full thermo-physical behaviour of rubbers, including the effects of the strain rate (sr=f(U)) and the cure degree (omega=ω) on the computation of the transport coefficients.

Finally, a new transport model called nonNewtonianRubberTransport has been implemented to describe the rubber viscosity acording to the “Reactive Viscosity Model”, Equation (20).

#### 2.3.1. Texture Wall Law: ROM

The effective viscosity wall law described in Section 2.2 is implemented in the OpenFOAM solver (rubberFoam) by means of a reduced order model (ROM), aimed at describing the behaviour of a complex system using simple mathematical expressions without losing relevant information.

An in-house developed ROM-generation algorithm based on Canonical Polyadic Decomposition (CPD) of tensors [28,29] is used in this work. It is able to transform a function of *N* not necessarily independent variables into the product of *N* one-dimensional functions. Each of these *N* one-dimensional functions takes one argument only, so that the number of one-dimensional functions is the same as the number of the system’s variables. This can be written as follows:

(31) $$F_{\left(v_{1},\ldots ,v_{N}\right)}=\sum_{m=1}^{M}\omega_{m}\prod_{n=1}^{N}{f_{\left(m,n\right)}}_{\left(v_{n}\right)}$$

where *M* is the order of approximation of the ROM and ω are weighting coefficients.

According to Equation (29), the effective viscosity of the rubber at the textured wall depends on $\tau_{wall_{\left(T\right)}}^{{}^{\prime }},U_{p},y_{P}$ and *T*, where $\tau_{wall_{\left(T\right)}}^{{}^{\prime }}$ can be numerically calculated under different operating conditions $U_{p},y_{P}$ and *T* (see Section 3.1) and later modelled as, from Equation (31):

(32) $$\tau_{wall_{\left(U_{P},y_{P},T\right)}}^{{}^{\prime }}=\sum_{m=1}^{M}\omega_{m}{f_{\left(m,U_{P}\right)}}_{\left(U_{P}\right)}{f_{\left(m,y_{P}\right)}}_{\left(y_{P}\right)}{f_{\left(m,T\right)}}_{\left(T\right)}$$

The output of the ROM is a text file (ROM_output_file.txt, see Appendix A) containing the values of all the parameters involved in Equation (32), which is stored in the constant folder of the OpenFOAM case. The rubberFoam solver reads it and calculates the viscous stress tensor at each time-step and for each face of the textured wall boundaries (modeled as smooth walls), given the resulting $U_{P}$, $y_{P}$ and *T* at the corresponding cell near the wall face.

Finally, the viscosity of the rubber (Equation (20)) on every face of the textured wall boundaries is replaced by the corresponding effective viscosity:

(33) $$\mu_{wall_{{}_{\left(T\right)}}}^{eff}=\left(y_{P}/U_{P}\right)\tau_{wall_{\left(T\right)}}^{{}^{\prime }}$$

### 2.4. Experimental Rubber Characterization

The selected rubber is a fluorocarbon elastomer whose hardness is 80 Shore A, denominated FKM80A, whose characterization is performed experimentally [30] and consists of: (i) Rubber-Capillary-Rheometer (RCR) experiments at 80, 100 and 120 °C from 10 s^−1^ up to 750 s^−1^ shear rate, the resulting data are used to fit the parameters of the “Reactive Viscosity Model” (Equation (20) reported in Table 1); and (ii) Moving Die Rheometer (MDR) tests at four different temperatures (160, 170, 180 and 190 °C) to estimate the value of the cure reaction-kinetics parameters (Equation (24) reported in Table 2). RCR and MDR raw data are attached as Supplementary Materials. Besides, the thermal conductivity (0.413 W/(m K)) and the specific heat capacity (0.86 J/(g K)) are obtained from hot disk and a DSC experiments respectively.

## 3. Results

In this section the methodology presented in this paper is applied to analyze the influence of a given wall texture pattern on the rubber filling dynamics focusing on one process variable, the injection pressure.

**Figure 2:**
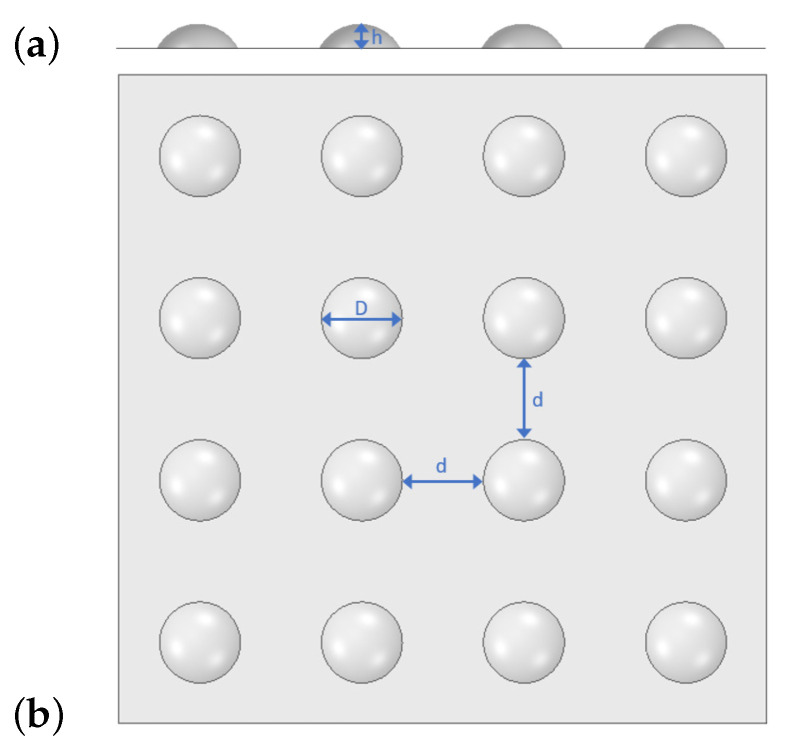
Wall texture—Dimples (D = 100 μm, h = 30 μm, d = 100 μm): (**a**) profile view; (**b**) plan view.

The texture considered in this study consists of dimples of 100 μm in diameter (D), 30 μm in height (h) and 100 μm spacing, as is illustrated in Figure 2.

Section 3.1 presents the numerical characterization of that wall texture for the given rubber (see Section 2.4), a prior step to apply the methodology. Then, Section 3.2 presents the results of the methodology for two different cases: (i) filling of a fully textured cylindrical straight pipe; and (ii) filling of an industrial mould whose walls are partially textured.

### 3.1. Rubber Flow Near the Textured Wall

To implement the effective wall viscosity on the macroscopic-scale simulation as described in Section 2.3.1, it is necessary to study the flow close to the textured wall depicted in Figure 2 and generate the dataset required to build the ROM model, Equation (32). To this end, 3D simulations of the rubber flow within a representative volume have been performed for different driving forces (in the range of 2.5 to 10 MPa/m) and temperatures (450–473 K).

**Figure 3:**
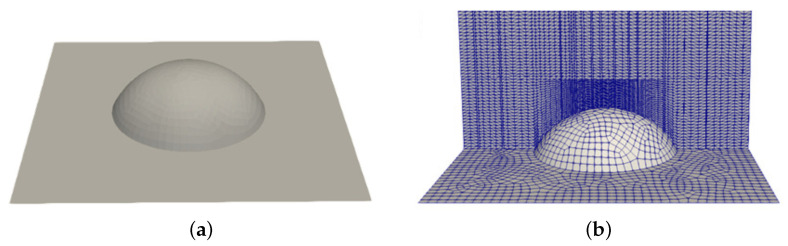
Representative domain near the wall: (**a**) geometry of the base (200 μm × 200 μm) with a dimple (D = 100 μm; h = 30 μm); (**b**) mesh close to the dimple at the base.

The representative volume considered in this study, sketched in Figure 1, is an hexahedron (200 μm × 200 μm × 500 μm), whose base stands for the textured wall and includes a dimple being centrally placed. From a preliminary study, where the volume height was 3850 μm (radii of the textured channels in the D-ring mould—see Section 3.2.2), it was concluded that the dimple-induced flow perturbations are restrained to a thin layer close to the wall and therefore it is suitable to reduce the domain height to 500 μm and set the fluid velocity at the upper face to the corresponding velocity profile obtained for the non-textured case at that height. Figure 3 shows the domain base together with some details of the corresponding mesh.

A set of eight cases is run according to the conditions specified in Table 3. Both temperature and pressure difference ranges correspond with those expected in the industrial case (Section 3.2.2). It must be noted that the ROM prediction outside these operating ranges is not applicable. Hence, the limits of these ranges were defined based on the results of the non-textured case macroscopic simulation (Section 3.2.2).

A summary of the boundary conditions used to simulate the representative volume near the wall, assuming periodic flow, is shown in Table 4. The velocity field obtained at the outlet is mapped to the inlet to include the effect on the inlet velocity profile of the presence of other dimples upstream. According to this set-up the flow driving force is the pressure drop along the domain, Δp. Since the representative volume is very small, the cases are considered isothermal and monophasic (e.g fully filled). Moreover, cure degree is assumed to remain zero (e.g., filling time < induction time).

**Figure 4:**
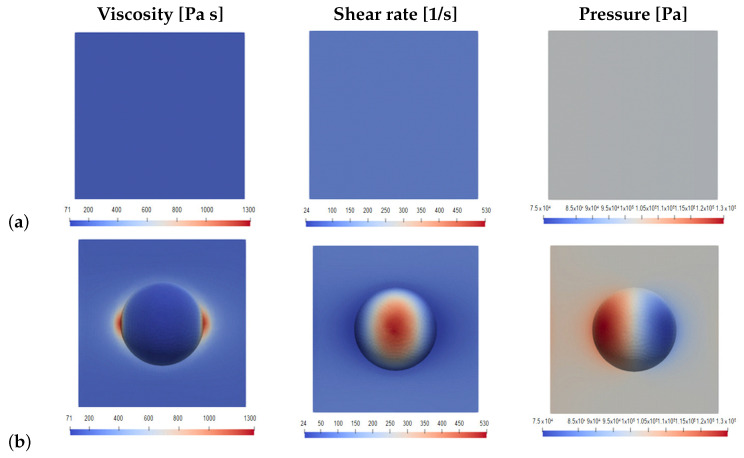
Flow on a representative domain near the wall, contours of viscosity, shear rate and pressure on the domain base (@ *T* = 450K & Δp = 1000 Pa): (**a**) smooth wall; (**b**) textured wall.

As an example, Figure 4 illustrates some results of this numerical study: viscosity, shear rate and pressure contours on the domain base wall. From the figure, comparing the contours resulting for the smooth wall (Figure 4a) with those of the textured wall (Figure 4b), the flow disturbance introduced by the dimple on the flow near the wall is clearly observed.

**Figure 5:**
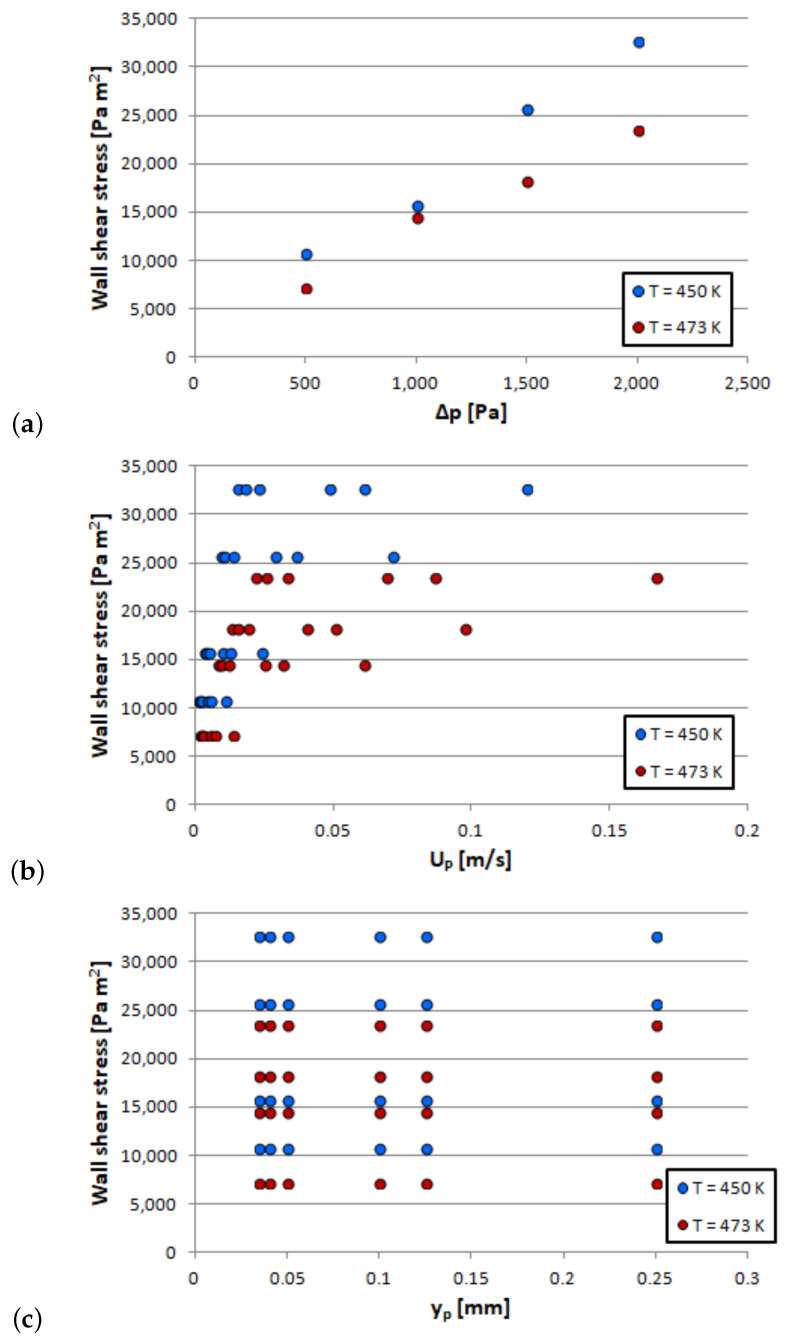
Flow on a representative domain near the wall, results: (**a**) Δp vs wall shear stress; (**b**) $U_{P}$ vs wall shear stress; and (**c**) $y_{P}$ vs wall shear stress.

The simulation results are further post-processed so that the viscous shear stress on the textured wall ($\tau_{wall_{\left(T\right)}}^{{}^{\prime }}$) is calculated together with the mean velocity of the flow ($U_{P}$) at different distances to the wall ($y_{P}$) for the corresponding operating temperature (*T*). The results are shown in Figure 5, the range of each parameter being: $U_{P}\in \left[0.0013,0.1666\right]$; $y_{P}\in \left[0.000035,0.00025\right]$; and T∈[450,473]. These data are eventually used to generate the ROM model that will predict the viscous shear stress at the textured wall according to Equation (32). Then the effective viscosity of the rubber at the wall will be calculated according to Equation (33). The resulting ROM model is used in the two cases presented in Section 3.2.

### 3.2. Rubber Injection in Textured-Wall Moulds

In this section, the OpenFOAM solver developed in Section 2.3, rubberFoam, is applied to simulate rubber injection in textured moulds in two different cases. First, as proof of concept, the rubber filling of a cylindrical straight pipe is considered (Section 3.2.1). Next, the methodology is applied to the simulation of the rubber injection on an industrial mould for the manufacturing of D-ring seals (Section 3.2.2).

#### 3.2.1. Test Case: Straight Pipe

The OpenFOAM solver rubberFoam is tested on a simple geometry, such as the case of a straight pipe, to analyze and quantify the influence of the micro textured wall on the filling process, specifically on the injection pressure. To this end, the following numerical study has been performed.

**Figure 6:**
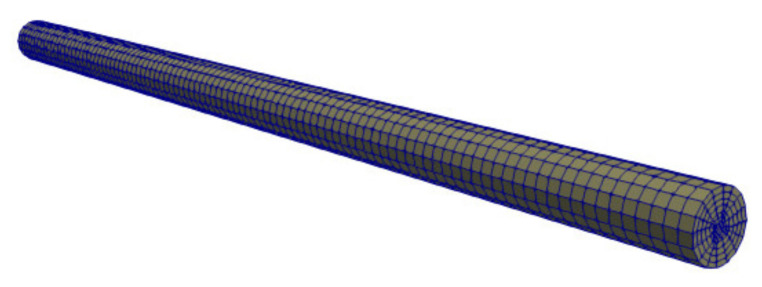
Filling of a straight pipe: numerical domain (case 3).

The numerical domain considered in this study is shown in Figure 6. It consists of a straight pipe of length *L* and diameter *D*, the magnitudes of which are two of the parameters accounted for in this study (see Table 5). It is worth mentioning that the dimples on the textured wall (Figure 2) are not included in the geometry, since their effect on the flow dynamics is modelled with the ROM model calculated in Section 3.1.

The domain is discretized to obtain an hexahedral computational mesh such as the one shown in Figure 6. During the meshing process, particular attention is paid to ensure that: (i) the distance between the wall and the center of the cell near the wall is between the corresponding limits of application of the texture wall law ($y_{P}\in \left[0.000035,0.00025\right]$); and (ii) keep the face size of the textured wall equal to or larger than the size of the base of the representative volume used to create the ROM model (0.2 mm × 0.2 mm).

Table 5 describes the cases considered in this study, considering the following parameters: (i) pipe length (*L*), (ii) pipe diameter (*D*) and (iii) filling flow rate ($U_{inlet}$). For cases 1 to 3, the geometry is the same and the inlet velocity varies; in cases 4 to 6, the influence of the pipe diameter is explored, while length and inlet velocity remain constant; lastly, cases 7 to 9, the impact of increasing length, with constant inlet velocity and diameter, is studied. It should be noted that cases 3, 5 and 7 are the same, so a total of seven cases are simulated.The case boundaries are defined by a uniform constant pressure at outlet (1 bar) and a uniform constant velocity at inlet ($U_{inlet}$), the wall is assumed to be non-slip and the system is considered isotherm (at 450 K).

**Figure 7:**
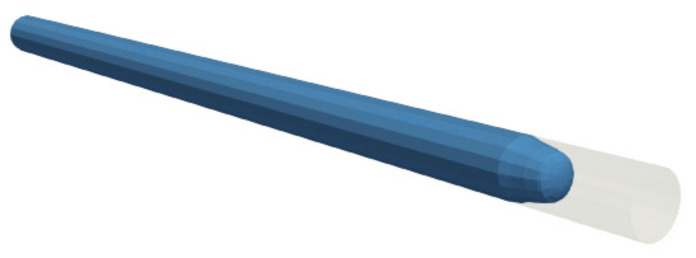
Filling of a straight pipe: rubber front at 90% filling time (case 3—textured wall).

**Figure 8:**
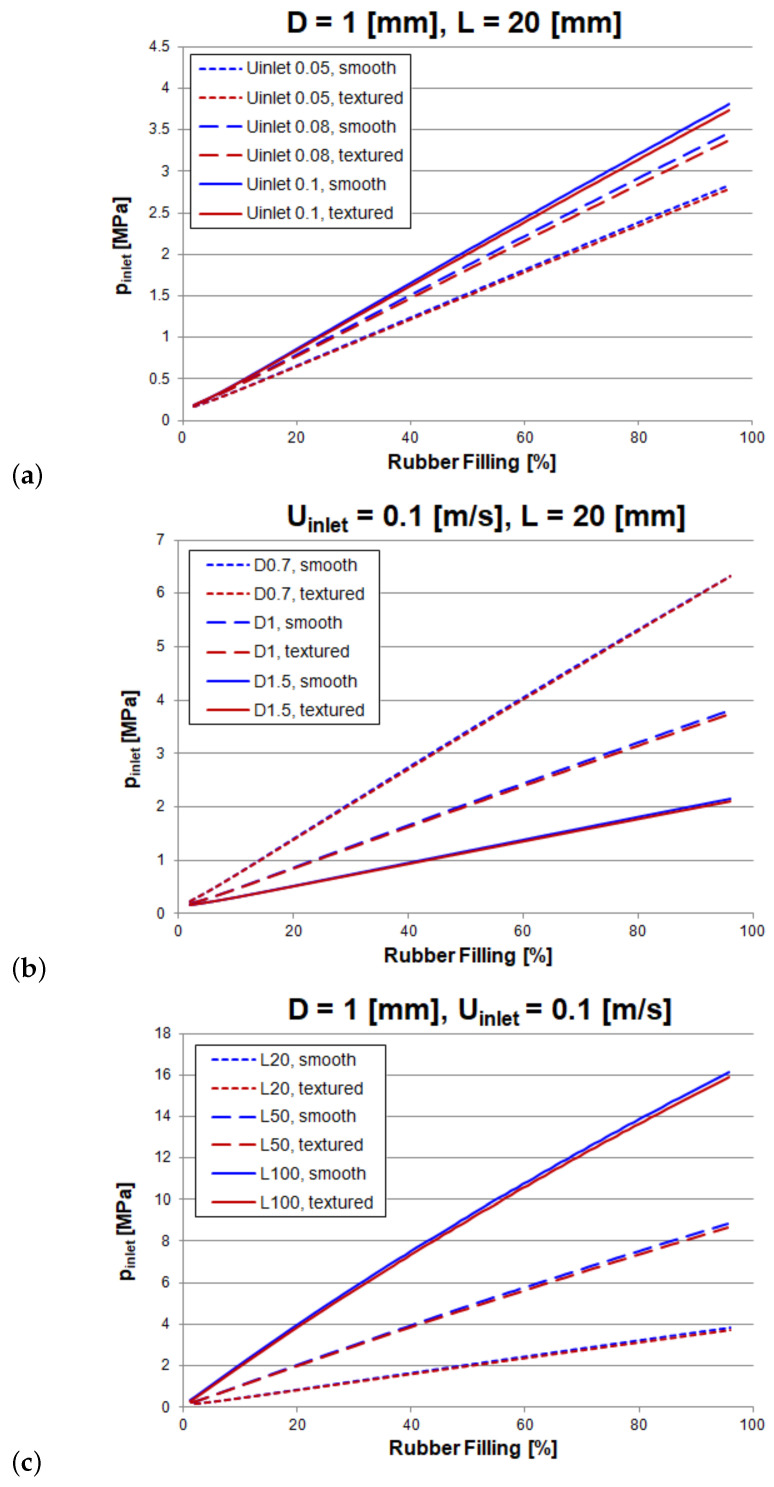
Results of the filling of a straight pipe (smooth vs textured wall): (**a**) influence of inlet velocity; (**b**) influence of diameter; (**c**) influence of length.

In order to study the effect of the textured wall on the inlet pressure (i.e., injection pressure), the cases reported in Table 5 are simulated twice using the solver rubberFoam: (1) with textured walls; and (2) with smooth walls, henceforth named textured and smooth respectively. The results are shown in Figure 7 and Figure 8.

Figure 7 illustrates the rubber front profile, which shows a central plug flow where the deformation rate is lower, while the layer closer to the wall experiences a higher shearing. This represents the characteristic behaviour of shear-thinning fluids, where the viscosity increases with decreasing shear rate towards the centre of the pipe.

From Figure 8, it can be concluded that injection pressure is, at any case, slightly larger for the smooth wall case than for the textured one regardless of the magnitude of the main system parameters: *L*, *D* and $U_{inlet}$. This means that the wall texture is not increasing the pressure drop in the system. On the contrary, wall texture slightly decreases wall friction.

A deeper analysis of the results plotted in Figure 8 leads to the following conclusions: (i) for a pipe of 1 mm in diameter and 20 mm in length, Figure 8a, the average inlet-pressure of textured case is about 2% smaller than the smooth case at any flow rate; (ii) for pipe diameters smaller than 1 mm, Figure 8b, no difference between smooth and textured wall is found whereas for larger diameters the injection pressure for the textured case is again around 2% lower than the smooth case; and (iii) the pipe length do not modify the former findings, Figure 8c, for a given pipe of 1mm in diameter the pressure loss difference remain around 2% no matter the length of the pipe (20, 50, 100 mm).

It is worth mentioning that case 4 is the only case where no difference is found between textured and smooth cases. It stands for the case with smaller diameter and the result could thus be affected by the meshing restrictions imposed by the methodology. For this particular case, where the texture size (*D* = 0.1 mm) is of the same order of magnitude than the pipe size (*D* = 0.7 mm) the methodology might be inappropriate.

#### 3.2.2. Real Case: D-Ring Mould

In the present section, the influence of the wall texture on an industrial mould is studied to check whether the conclusions reached in Section 3.2.1 still stand for a real industrial application. In particular, the injection process of rubber in a D-ring seal mould is numerically assessed for the case of a standard mould (smooth walls) and for a novel mould, where only the internal face of the D-ring is textured.

**Figure 9:**
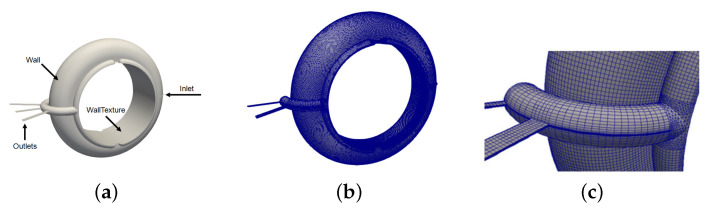
Mould cavity of a D-ring seal: (**a**) geometry; (**b**) mesh; and (**c**) mesh detail close to exit vents.

Figure 9a shows the geometry of the use case, i.e., a mould cavity for the manufacturing of D-ring seals. It has one inlet, three exit vents (“Outlets”), an internal face where texture can be applied (“WallTexture”) and an external smooth face (“Wall”).

Due to the D-ring symmetry the computational domain is reduced to the half and then discretized according to the mesh requirements imposed by the methodology (texture wall law): (i) the distance between the wall and the center of the cell near the wall is kept between the corresponding limits of application of the texture wall law ($y_{P}\in \left[0.000035,0.00025\right]$); and (ii) the face size of the textured wall is kept equal to or larger than the size of the base of the representative volume used to create the ROM model (0.2 mm × 0.2 mm). Figure 9b,c show the resulting computational domain, together with some details of the mesh near the outlet vents.

This numerical study consists of the simulation using the rubberFoam of two cases: (1) standard mould where all walls are smooth; and (2) textured mould, where the internal face is textured according to the pattern shown in Figure 2. Based on industrial experience, a filling time of 1 s is specified, and the average injection velocity is calculated accordingly and set as inlet boundary condition. A constant and uniform pressure field (1 bar) is defined at the outlets. Injection temperature is set to 450 K. Table 6 summarises the most relevant boundary conditions. Please note that the solver will automatically activate the texture wall law for viscosity at those boundaries whose name starts with wallTexture. Thus, the only difference between smooth and textured cases is that the internal ring wall is called wall and wallTexture respectively.

**Figure 10:**
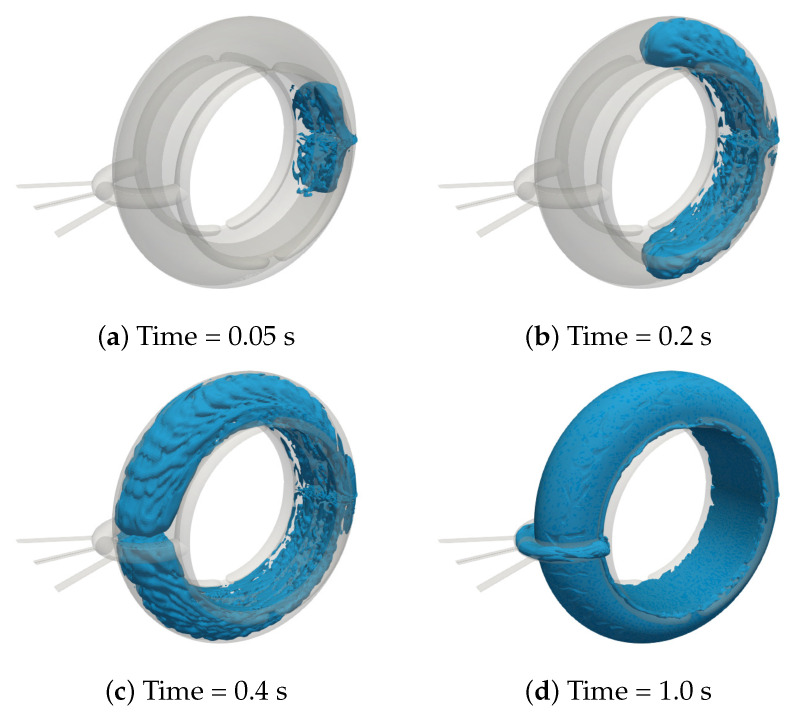
D-ring mould filling: Rubber front at different times, namely: (**a**) 0.05 s (**b**) 0.2 s (**c**) 0.4 s (**d**) 1 s.

The results of the numerical simulation of the mould filling process are illustrated in Figure 10, for the case of the standard mould (no texture). No significant differences are found for the novel mould (partially textured). From the figure, it is highlighted that such a detailed simulation allows the accurate visualization of the rubber front evolution. For instance, cavities where air is trapped are easily identified and weld lines on the piece surface can be predicted. Moreover, unexpected flow dynamics can be visualised such as the fact that, due to the very high injection velocities, the rubber front reaches the surroundings of the mould oultet in less than 0.4 s, when only 36% of the mould is actually filled.

**Figure 11:**
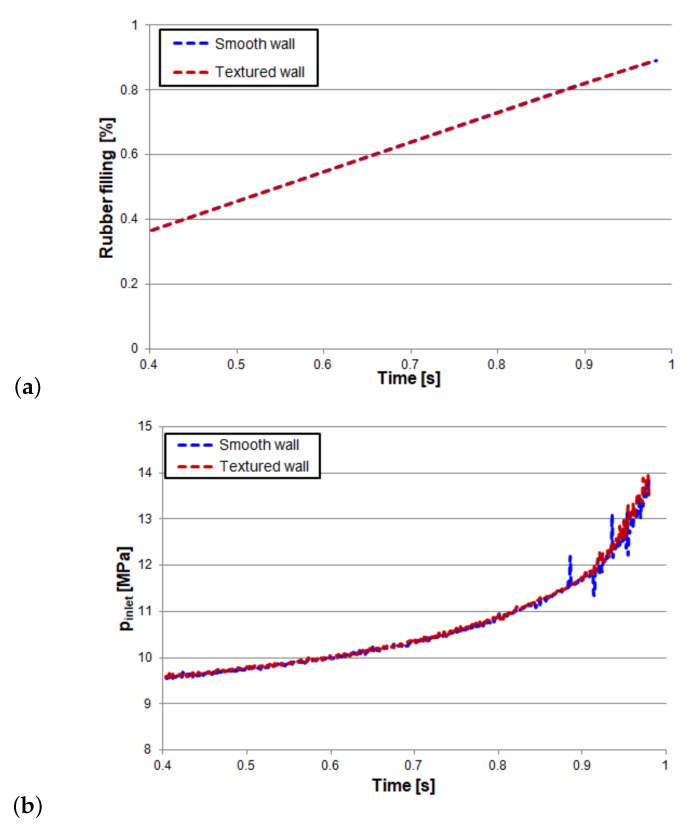
D-ring results:(**a**) Rubber filling rate and (**b**) Inlet pressure evolution in time.

The evolution of the inlet pressure as the rubber filling proceeds is plotted in Figure 11. As expected, the rubber filling rate of the textured case does not differ from the smooth case, because the inlet flow rate is equal and constant in both cases. Regarding the inlet pressure, the differences between the smooth and textured cases are almost negligible.

## 4. Discussion

In this numerical study the influence of the wall texture of the rubber injection moulds on the operating injection pressure is assessed. A priori, one might expect higher pressure drops in textured walls due to the increase of effective area and a higher resistance to flow induced by the dimples (i.e., higher wall rugosity). However, the results of this study show that this effect can be counterbalanced by the non-Newtonian behaviour of the rubber, whose viscosity decreases near the textured wall due to the larger shear stress induced by the texture, illustrated in Figure 4. These two effects seem to almost cancel each other out and the macroscopic flow is thus not significantly changed.

The results of the rubber filling of a textured straight pipe, reported in Section 3.2.1, show that the textured case requires less inlet pressure than its corresponding smooth case, because the wall texture decreases the effective viscosity of the rubber at the wall. These variations are small (around 2%) but are persistent across all tested cases, independently of the selected length, diameter (>1 mm) and inlet velocity. This is an outstanding result that supports the use of textured wall moulds for rubber injection, since it can improve the final component properties (e.g., rubber seal friction) and the manufacturing process. Although a 2% reduction stands for a small percentage, it can result in a significant net reduction of energy consumption during the process, and so of the ${\mathrm{CO}}_{2}$ emissions, given the fact that seals are components that in most cases are produced in very high volumes (thousands or millions), and that there is a huge variety of seals used in a wide variety of industrial components.

In Section 3.2.2, the rubber injection for the manufacturing of D-ring seals is numerically assessed both for a standard mould (non-textured) and a novel one (partially textured). The results being in agreement with the conclusions reached for the simple case of the straight pipe (Section 3.2.1), as it is found that the textured wall do not increase the inlet pressure. It is thus proved that for a real application textured wall moulds could be advantageous to enhance the piece’s properties without hindering the manufacturing process. It is worth mentioning that the positive effect of the texture found in Section 3.2.1, i.e., a pressure drop reduction of around 2%, is not seen for the real application. This could be explained by the fact that here only a small part of the mould area is textured, whereas the straight pipe considered in Section 3.2.1 is fully textured.

The numerical analysis presented in this paper focuses on a particular texture described in Figure 2 and a particular rubber (FKM80A, Table 1). As a future work, the methodology implemented and deployed in this research could be used to analyze the effect of other types of textures or other rubbers.

## 5. Conclusions

A novel numerical methodology for the assessment of the micro-textured wall effects on rubber injection moulding is presented in this paper. The methodology is fully based on two open-sources tools: (a) OpenFOAM, a standard open-source CFD platform [10]; and (b) Twinkle a reduced order model builder library [28,29]. As a result, a new and advanced OpenFOAM solver, rubberFoam was developed to calculate the rubber flow during rubber injection on textured moulds, where the effective viscosity at the wall corresponding to the textured surface is introduced by means of a reduced order model. The solver is then tested in both an academic case (cylindrical straight pipe) and an industrial case (D-ring seal mould). At any case, the textured walls do not induce any negative effect on the manufacturing process, as the injection pressure remains equal or even decreases around 2% with respect to the reference case (no textured walls). This behaviour is due to the non-Newtonian nature of the rubber, as the viscosity near the wall decreases due to the higher shear rates induced by the wall texture. The results of this study support the current trend towards mould walls texturization to improve the pieces mechanical properties, without compromising (or even enhancing) the manufacturing process or production costs.

## Figures and Tables

**Table 1:** Rubber characterization: Parameters of the “Reactive Viscosity Model”.

Parameter	Value
*B*	0.250839
$T_{b}$	9947.615
$\tau^{*}$	0.027699
*n*	0.461665
$\omega_{g}$	0.796923
$C_{1}$	4.291385
$C_{2}$	−4.13821

**Table 2:** Rubber characterization: Parameters of rubber cure kinetics.

Parameter	Value
$A_{1}$	3.77 × $10^{11}$
$E_{1}$	13,350
$A_{2}$	8.72 × $10^{13}$
$E_{2}$	13,962
*m*	1.22
*n*	1.36
$B_{1}$	1.05 × $10^{-7}$
$B_{2}$	9124.8

**Table 3:** Microscale model: case definitions.

T [K]	Δp [Pa]
450	500
	1000
	1500
	2000
473	500
	1000
	1500
	2000

**Table 4:** Microscale model: boundary conditions.

Patch	Type	Relevant Boundary Definition
Inlet (−x)	mappedPatch	Pressure: fixedValue Velocity: mappedField
Outlet (+x)	patch	Pressure: fixedValue
Lateral wall (−y)	symmetry	Symmetry
Lateral wall (+y)	symmetry	Symmetry
Upper wall (+z)	wall	Velocity: non-texturized case
Lower wall (−z)	wall	Velocity: No slip (0 0 0)

**Table 5:** Straight pipe: case definitions.

Cases	$U_{\mathrm{inlet}}$ [m/s]	D [mm]	L [mm]
1	0.05	1	20
2	0.08		
3	0.1		
4	0.1	0.7	20
5		1	
6		1.5	
7	0.1	1	20
8			50
9			100

**Table 6:** D-ring: boundary conditions.

Patch	Type	Relevant Boundary Definition
inlet	patch	Velocity: flowRateInletVelocity
outlet_1, outlet_2	patch	Pressure: fixedValue
symmetry	symmetry	symmetry
wall	wall	Velocity: noSlip
wallTexture	wall	Velocity: noSlip
		Viscosity: Texture wall law

## Data Availability

Experimental data resulting from the rubber characterization tests are attached as Supplementary Material.

## Supplementary Materials

Supplementary materials are available online at https://www.mdpi.com/article/10.3390/polym13111739/s1.

## Appendix A. ROM_Output_file.txt

The following text file stands for the output of the ROM builder. This text file contains the values of the functions and weighting factors of Equation (32). The new OpenFOAM solver (rubberFoam) reads this file and calls a function (evalROM), available at the ROM builder library [28,29], which interprets the data and evaluates Equation (32) to predict the viscous shear tensor at the textured wall.

***********************************************************************
*** ROM summary
*** Number of terms: 5
*** Number of dimensions: 3
*** Dimension 1 discretization: 2 2 2 2 2
*** Dimension 2 discretization: 8 8 8 8 8
*** Dimension 3 discretization: 12 12 12 12 12
***********************************************************************
ROM Data:
Term 1
Alpha: 412848
Dimension 1
450 0.853266 473 0.521476
Dimension 2
3.5 × 10${}^{-5}$ 0.536588 4.39583 × 10${}^{-5}$ 0.475352 5.29167 × 10${}^{-5}$ 0.435076 9.77083 × 10${}^{-5}$ 0.381214 0.000106667 0.131042 0.000124583 0.299927
0.000133542 0.0146306 0.00025 0.210134
Dimension 3
0.00136384 0.0608335 0.00824975 0.132693 0.0151356 0.170953 0.0220216 0.203596 0.0289075 0.229498 0.0357934 0.252525 0.0426793 0.276441
0.0495652 0.294166 0.0564511 0.31351 0.063337 0.327378 0.0908806 0.391973 0.166625 0.51915
Term 2
Alpha: 2667.23
Dimension 1
450 −0.943357 473 0.33178
Dimension 2
3.5 × 10${}^{-5}$ 0.0138087 4.39583 × 10${}^{-5}$ −0.354879 5.29167 × 10${}^{-5}$ −0.890803 9.77083 × 10${}^{-5}$ 0.0510481 0.000106667 0.0175478 0.000124583
−0.114979 0.000133542 −0.00560872 0.00025 0.253328
Dimension 3
0.00136384 −0.168722 0.00824975 0.816757 0.0151356 −0.286778 0.0220216 −0.0292196 0.0289075 −0.131955 0.0357934 −0.138186 0.0426793
0.330298 0.0495652 −0.0797557 0.0564511 0.160481 0.063337 −0.093528 0.0908806 0.18641 0.166625 0.011386
Term 3
Alpha: 1645.94
Dimension 1
450 0.798857 473 0.601521
Dimension 2
3.5 × 10${}^{-5}$ −0.25005 4.39583 × 10${}^{-5}$ 0.157966 5.29167 × 10${}^{-5}$ 0.0714464 9.77083 × 10${}^{-5}$ 0.868813 0.000106667 0.298654 0.000124583
0.217874 0.000133542 0.010628 0.00025 0.125718
Dimension 3
0.00136384 0.623631 0.00824975 0.0585777 0.0151356 −0.631778 0.0220216 −0.255556 0.0289075 −0.00579108 0.0357934 −0.0640496 0.0426793
0.0493176 0.0495652 −0.0170427 0.0564511 0.252543 0.063337 −0.111981 0.0908806 0.110066 0.166625 0.218881
Term 4
Alpha: 2189.93
Dimension 1
450 −0.652909 473 −0.757436
Dimension 2
3.5 × 10${}^{-5}$ 0.0795748 4.39583 × 10${}^{-5}$ 0.824329 5.29167 × 10${}^{-5}$ −0.537909 9.77083 × 10${}^{-5}$ 0.0884352 0.000106667 0.0303996 0.000124583
0.0814518 0.000133542 0.00397326 0.00025 −0.097
Dimension 3
0.00136384 −0.00741717 0.00824975 0.183312 0.0151356 0.137525 0.0220216 −0.756504 0.0289075 0.535539 0.0357934 −0.174232 0.0426793
0.0858151 0.0495652 −0.0884338 0.0564511 −0.0504824 0.063337 0.0901165 0.0908806 0.177468 0.166625 −0.0249437
Term 5
Alpha: 1087.76
Dimension 1
450 −0.353853 473 0.935301
Dimension 2
3.5 × 10${}^{-5}$ −0.345931 4.39583 × 10${}^{-5}$ −0.171289 5.29167 × 10${}^{-5}$ 0.914857 9.77083 × 10${}^{-5}$ −0.0957467 0.000106667 −0.0329129 0.000124583
0.00318454 0.000133542 0.000155344 0.00025 0.0613852
Dimension 3
0.00136384 0.139599 0.00824975 0.33411 0.0151356 −0.215309 0.0220216 0.0686149 0.0289075 0.00205378 0.0357934 −0.0832903 0.0426793
0.0700626 0.0495652 −0.451532 0.0564511 −0.679474 0.063337 0.363554 0.0908806 0.0879399 0.166625 0.022273
