# Supplementary material for: Numerical Approach for the Assessment of Micro-Textured Walls Effects on Rubber Injection Moulding
Source: Polymers (Basel). 2021 May 26;13(11):1739. doi: 10.3390/polym13111739 (PMC8198524; doi:10.3390/polym13111739)

FKM80 – MOVING DIE RHEOMETER DATA

Temperature							
160ºC		170ºC		180ºC		190ºC	
Time [min]	S' [dNm]	Time [min]	S' [dNm]	Time [min]	S' [dNm]	Time [min]	S' [dNm]
0.030	3.370	0.035	4.874	0.032	3.740	0.045	3.701
0.060	4.637	0.065	4.601	0.062	4.070	0.075	3.796
0.090	4.589	0.095	3.783	0.092	3.255	0.105	2.785
0.120	3.761	0.125	3.023	0.122	2.570	0.135	2.166
0.150	3.153	0.155	2.527	0.152	2.141	0.165	1.804
0.180	2.745	0.185	2.204	0.182	1.868	0.195	1.587
0.210	2.474	0.215	1.985	0.212	1.682	0.225	1.457
0.240	2.293	0.245	1.840	0.242	1.555	0.255	1.374
0.270	2.166	0.275	1.746	0.272	1.476	0.345	1.309
0.300	2.081	0.365	1.635	0.362	1.391	0.355	1.310
0.390	1.970	0.432	1.623	0.392	1.392	0.442	1.420
0.427	1.954	0.445	1.622	0.429	1.405	0.525	1.641
0.510	1.938	0.575	1.658	0.512	1.462	0.615	2.034
0.530	1.941	0.829	1.819	0.692	1.723	0.663	2.309
0.570	1.948	0.935	1.913	0.826	2.040	0.675	2.389
0.824	2.024	1.226	2.241	0.842	2.083	0.705	2.602
1.221	2.181	1.295	2.331	0.937	2.391	0.787	3.309
1.470	2.278	1.479	2.622	0.962	2.485	0.821	3.654
1.618	2.348	1.623	2.887	1.082	2.999	0.839	3.856
2.015	2.544	1.625	2.891	1.157	3.391	0.855	4.045
2.160	2.637	1.895	3.518	1.219	3.745	0.885	4.422
2.412	2.810	1.933	3.622	1.222	3.764	0.915	4.827
2.582	2.938	2.020	3.874	1.232	3.818	0.945	5.266
2.730	3.060	2.059	3.991	1.352	4.640	0.975	5.737
2.809	3.128	2.135	4.238	1.472	5.614	1.005	6.240
3.205	3.530	2.375	5.139	1.592	6.763	1.035	6.771
3.270	3.600	2.417	5.311	1.619	7.053	1.065	7.332
3.533	3.934	2.585	6.088	1.712	8.081	1.095	7.915
3.536	3.938	2.795	7.210	1.832	9.564	1.125	8.525
3.602	4.027	2.814	7.318	2.016	12.065	1.215	10.491
3.750	4.237	3.005	8.505	2.094	13.162	1.236	10.968
3.999	4.623	3.185	9.744	2.192	14.486	1.325	13.032
4.142	4.872	3.210	9.932	2.282	15.645	1.395	14.641
4.396	5.352	3.425	11.557	2.372	16.682	1.425	15.294
4.502	5.574	3.607	13.005	2.413	17.117	1.455	15.921
4.793	6.231	3.665	13.463	2.462	17.598	1.485	16.517
4.862	6.401	3.875	15.113	2.552	18.380	1.515	17.081
5.190	7.275	4.004	16.056	2.672	19.237	1.545	17.602
5.192	7.279	4.087	16.621	2.792	19.935	1.575	18.083
5.582	8.470	4.267	17.743	2.810	20.027	1.633	18.903
5.587	8.488	4.401	18.474	2.942	20.629	1.635	18.933

5.912	9.615	4.417	18.554	3.122	21.279	1.665	19.302
5.984	9.885	4.567	19.248	3.207	21.534	1.695	19.636
6.182	10.641	4.798	20.134	3.302	21.794	1.785	20.471
6.381	11.445	5.047	20.898	3.542	22.346	1.875	21.100
6.497	11.919	5.195	21.286	3.604	22.460	1.965	21.604
6.692	12.738	5.347	21.645	3.665	22.576	2.030	21.899
6.778	13.108	5.592	22.138	3.872	22.901	2.085	22.112
7.175	14.817	5.647	22.236	4.001	23.068	2.171	22.408
7.322	15.428	5.989	22.779	4.323	23.429	2.235	22.593
7.572	16.432	6.093	22.935	4.398	23.498	2.427	23.035
7.622	16.616	6.097	22.940	4.795	23.803	2.445	23.072
7.892	17.570	6.386	23.294	4.893	23.869	2.715	23.484
7.969	17.814	6.637	23.563	5.192	24.036	2.824	23.611
8.193	18.500	6.783	23.714	5.589	24.220	3.105	23.873
8.366	18.970	7.117	23.987	5.673	24.258	3.220	23.953
8.523	19.369	7.180	24.036	5.986	24.365	3.585	24.167
8.760	19.903	7.577	24.310	6.383	24.479	3.617	24.176
8.763	19.908	7.597	24.315	6.780	24.576	4.014	24.323
8.793	19.973	7.974	24.540	6.813	24.584	4.411	24.434
9.159	20.681	8.371	24.746	7.176	24.648	4.487	24.454
9.556	21.329	8.408	24.764	7.573	24.711	4.808	24.515
9.933	21.861	8.768	24.915	7.970	24.768	5.205	24.577
		9.164	25.059	8.315	24.803	5.602	24.618
		9.488	25.167	8.367	24.805	5.687	24.630
		9.561	25.195	8.764	24.843	5.999	24.658
		9.938	25.298	9.161	24.869	6.396	24.683
				9.558	24.902	6.793	24.699
				9.855	24.916	7.190	24.713
				9.935	24.925	7.587	24.724
						7.984	24.733
						8.148	24.739
						8.381	24.740
						8.778	24.741
						9.174	24.740
						9.571	24.736
						9.618	24.742
						9.778	24.752
						9.948	24.738

Temperature:	160°C		170°C		180°C		190°C	
S'@Max S' (MH)	21.9	dNm	25.3	dNm	24.93	dNm	24.75	dNm
S'@Min S' (ML)	1.94	dNm	1.62	dNm	1.39	dNm	1.31	dNm
Time@1 dNm	2.58	min	1.48	min	0.94	min	0.66	min
Time@2 dNm	3.54	min	1.93	min	1.16	min	0.79	min
Time@10% cure	3.53	min	2.06	min	1.22	min	0.82	min
Time@50% cure	6.5	min	3.66	min	2.09	min	1.32	min
Time@90% cure	8.76	min	6.09	min	3.66	min	2.17	min
S"@Max S"	2.77	dNm	3.05	dNm	2.98	dNm	3.2	dNm
S"@Min S"	2.13	dNm	1.88	dNm	1.65	dNm	1.59	dNm

MOVING DIE RHEOMETER CURVES

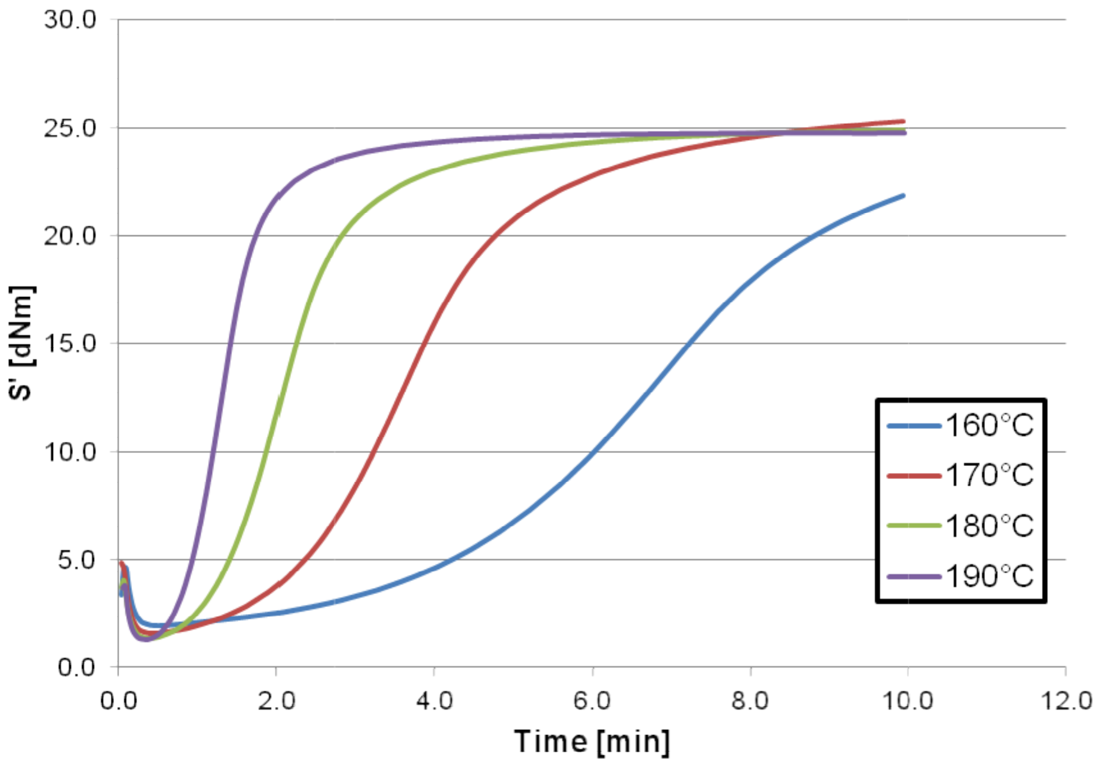

FKM80 – RUBBER CAPILLAR RHEOMETER DATA

Temperature	80°C	100°C	120°C
**Shear Rate[1/s]**		**Viscosity [Pa s]**	
**10**	9981.782	4597.109	2521.016
**25**	6610.328	2852.797	1656.715
**50**	3695.164	2114.117	1499.172
**100**	2440.609	1482.797	1187.402
**250**	1783.09	949.168	704.883
**500**	1098.978	838.75	514.672
**750**	874.326	633.041	437.307

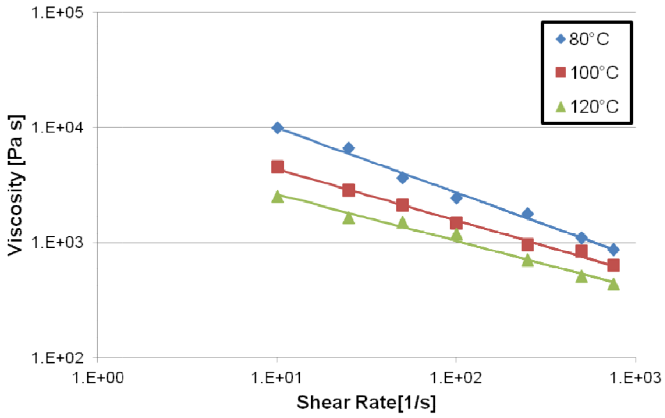

Supplement: Supplementary file 1 [file polymers-13-01739-s001.zip › polymers-1222966-supplementary.pdf]
